# Japanese Cohort Study and Meta‐Analysis Demonstrate Significant Association Between Serotonin Transporter‐Linked Polymorphic Region and Schizophrenia

**DOI:** 10.1002/npr2.70114

**Published:** 2026-03-29

**Authors:** Masao Miyachi, Toshiyuki Shirai, Shohei Okada, Kiriko Minami, Ryo Tsukamoto, Kentaro Mouri, Satoshi Okazaki, Ikuo Otsuka, Akitoyo Hishimoto

**Affiliations:** ^1^ Department of Psychiatry Kobe University Graduate School of Medicine Kobe Japan

**Keywords:** genetic, Japan, meta‐analysis as topic, polymorphism, schizophrenia, serotonin plasma membrane transport proteins

## Abstract

Schizophrenia is a prevalent psychiatric disease that substantially affects health and increases mortality risk. The 5‐hydroxytryptamine system has been implicated in its pathogenesis. The *SLC6A4* gene encodes the serotonin transporter, which regulates synaptic 5‐hydroxytryptamine through reuptake into presynaptic neurons. Within its promoter region, there is a 44‐base pair insertion/deletion polymorphism, known as serotonin‐transporter‐linked polymorphic region (5‐HTTLPR). We investigated the association between 5‐HTTLPR polymorphism and schizophrenia in the cohorts, including the largest sample size of Asian schizophrenia patients to date. We included 467 patients with schizophrenia and 361 controls, all of Japanese ancestry. We extracted DNA from peripheral blood samples. After amplification with PCR, we analyzed the association between 5‐HTTLPR genotypes and alleles and schizophrenia. Furthermore, we conducted a meta‐analysis with previous literatures. 5‐HTTLPR genotype distribution differed significantly between patients and controls (*p* = 0.007). The short allele was significantly more frequent in schizophrenia patients (odds ratio = 1.39 [95% Cl = 1.08–1.77], *p* = 0.007). The meta‐analysis demonstrated a significant association between the short allele and schizophrenia (odds ratio = 1.06 [95% Cl = 1.01–1.11], *p* = 0.024). This study demonstrates a significant association between 5‐HTTLPR polymorphism and schizophrenia. While further research is needed, the findings suggest 5‐HTTLPR may represent a promising target for future therapeutic strategies.

## Abstract 2

This study demonstrates a significant association between the 5‐HTTLPR polymorphism and schizophrenia risk. The short allele was significantly overrepresented in patients (OR = 1.39, *p* = 0.007), suggesting a potential contribution to schizophrenia risk.

## Graphical abstract

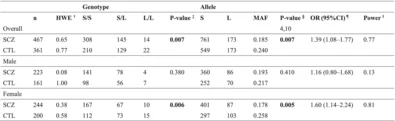

## 1 Introduction

Schizophrenia is a major psychiatric disease characterized by hallucinations, delusions, and disorganized thinking and behaviors. Although pharmacological treatments and psychological interventions for schizophrenia have continued to advance, a considerable proportion of patients continue to experience recurrent hospitalizations and long‐term impairments in social and occupational functioning [1]. Schizophrenia is common worldwide and imposes a heavy burden on individuals, including an elevated risk of premature mortality across the lifespan [2]. The 5‐hydroxytryptamine (5‐HT, serotonin) system has long been implicated in the neurodevelopmental and neurotransmitter‐based hypothesis of schizophrenia, as serotonergic signaling plays an important role in brain development and neural circuit modulation [3]. In this context, several antipsychotic agents exert part of their therapeutic effects through actions on 5‐HT receptors in the central nervous system, further highlighting the relevance of the serotonin system to schizophrenia [4]. The *SLC6A4* gene encodes the serotonin transporter (5‐HTT), which regulates serotonergic neurotransmission by reuptaking serotonin into presynaptic neurons [5]. Within the promoter regions of the *SLC6A4* gene, there is a 44‐base pair insertion/deletion polymorphism, which is well known as serotonin transporter‐linked polymorphic region (5‐HTTLPR). This polymorphism generates two major alleles: the long (L) allele and the short (S) allele [6]. The L allele is associated with higher 5‐HTT function, facilitating serotonin reuptake and termination of its synaptic action, whereas the S allele is linked to lower transcription of the *SLC6A4* gene and reduced transporter function [7].

Although some studies have examined the relationship between 5‐HTTLPR polymorphism and schizophrenia, their findings remain inconsistent, and no definitive association has been established [8]. These inconsistencies may be explained by the small sample sizes and limited statistical power of most previous studies. In particular, the number of studies conducted in Asian populations remains extremely limited compared with those in European populations, despite known ethnic differences in genetic architecture. In the present study, we investigated the association between 5‐HTTLPR polymorphism and schizophrenia with the cohort including the largest sample size of patients with schizophrenia among Asian cohort studies.

## 2 Methods

### 2.1 Participants

This study included 467 patients with schizophrenia and 361 healthy controls. All participants were of Japanese descent. Diagnoses of schizophrenia were established according to the criteria of the *Diagnostic and Statistical Manual of Mental Disorders*, Fourth Edition (DSM‐IV), or Fifth Edition (DSM‐5). Each patient was independently evaluated by at least two board‐certified psychiatrists to ensure diagnostic accuracy. For the healthy control group, inclusion required the absence of any current or past psychiatric disease and no family history of psychiatric diseases or substance dependence (excluding nicotine) in first‐degree relatives. These conditions were confirmed by at least two psychiatrists. All participants provided written informed consent, and the study protocol was approved by the institutional ethics committee of Kobe University.

### 2.2 Genotyping

We obtained peripheral blood samples from all patients with schizophrenia and healthy controls and stored them at −80°C. We extracted DNA from these samples using the QIAamp DNA Blood Midi Kit (Qiagen, Valencia, CA, USA) following the manufacturer's instructions. We examined the DNA concentration and purity using the NanoDrop spectrophotometer (Thermo Fisher Scientific, Wilmington, DE, USA). For all samples, we performed the polymerase chain reactions (PCR) to distinguish the L or S 5‐HTTLPR polymorphism. Each PCR assay volume (10 μL) contained 10 ng of genomic DNA, 5 μL of AmpliTaq Gold 360 Master Mix (Thermo Fisher Scientific), 10% GC enhancer, 0.1 μM, and 5 pmol of each forward and reverse primer.

Primer sequences were designed according to the method of Hu et al.: forward primer, 5′‐FAM‐GGCGTTGCCGCTCTGAATGC3′; reverse primer, 5′‐GAGGGACTGAGCTGGACAACCAC‐3′ [9]. We set the PCR thermal cycling conditions as follows: an initial denaturation step was performed at 95°C for 10 min, followed by 40 cycles of 95°C for 30 s, touchdown annealing from 63°C to 55°C for 30 s, and 72°C for 30 s. A final elongation step was performed at 72°C for 7 min. Amplified products were analyzed with the SeqStudio Genetic Analyzer (Applied Biosystems, Waltham, MA, USA) and the GeneMapper Software version 6 (Applied Biosystems). The PCR products of 529 and 486 base pair corresponded to the L and S alleles respectively, as previously reported [9]. Each participant was classified into one of three genotypes: SS, SL, or LL.

### 2.3 Statistical Analysis

All statistical analyses were performed using R version 4.4.2 (R Development Core Team, Vienna, Austria). Group differences in demographic characteristics were evaluated with Fisher's exact test for sex and Welch's *t*‐test for age. 5‐HTTLPR genotype distributions (SS, SL, and LL) were compared between patients with schizophrenia and controls using the Cochrane‐Armitage trend test. Allelic associations (S vs. L) were evaluated with Fisher's exact test. Subgroup analyses stratified by sex were also conducted to assess potential sex‐specific associations. Hardy–Weinberg equilibrium of genotype distributions was tested using the recursion equations described by Wigginton et al. [10] Statistical power was estimated according to Cohen's h.

### 2.4 Meta‐Analysis

We performed a meta‐analysis to synthesize evidence from previous studies. We systematically searched candidate articles via the PubMed up to March 1, 2025, using the terms (“serotonin transporter” OR “SERTPR” OR “SERT‐in2” OR “5‐HTTLPR” OR “SLC6A4”) AND (“schizophrenia”). This meta‐analysis protocol was not registered. Studies were eligible if they met the following criteria: (1) investigating the 5‐HTTLPR polymorphism (S or L allele) in both schizophrenia or schizophrenic patients and healthy controls; (2) independent from other included studies; (3) adequate and sufficient information for performing data synthesis, and (4) publication in English. Titles and abstracts were screened first, and full texts were reviewed as necessary. Non‐eligible studies were excluded according to these criteria. Two reviewers (M.M. and T.S.) independently assessed eligibility, and disagreements were resolved through discussion and, when necessary, with mediation by S.O.

We divided phenotypes into schizophrenia or schizophrenic patients versus controls. We conducted a meta‐analysis with the R package metafor using a fixed‐effect model and assessed the association between 5‐HTTLPR polymorphism and phenotype through OR [11]. Heterogeneity was evaluated with the *I* ^2^ statistic, interpreted as follows: 0%–40% might not be important; 30%–60% may indicate moderate heterogeneity; 50%–90% may indicate substantial heterogeneity; and 75%–100% considerable heterogeneity [12].

## 3 Results

### 3.1 Genotype and Allele Analyses

Demographic characteristics did not differ significantly between patients with schizophrenia and healthy controls in terms of sex or age (Table 1). Detailed data of the participants were listed in Table S1. Genotype analysis revealed a significant difference in the distribution of 5‐HTTLPR genotypes between patients and controls (Cochran‐Armitage trend test, *p* = 0.007). Allele analysis further demonstrated that S allele frequency was significantly higher in patients than in controls (OR = 1.39 [95% Cl = 1.08–1.77], *p* = 0.007) as the result of Fisher's exact test. In subgroup analyses stratified by sex, a positive association between the S allele and schizophrenia was observed in both females (OR = 1.60 [95% Cl = 1.14–2.24], *p* = 0.005) and males (OR = 1.16 [95% Cl = 0.80–1.68], *p* = 0.414). However, statistical significance was achieved only in females, and the effect size was larger in females than in males (Table 2).

**TABLE 1:** Demographic and clinical characteristics.

	Control	Schizophrenia	*p*
Number	361	467	
Male/Female	161/200	223/244	0.399^a^
Age, mean ± SD	53.5 ± 19.2	51.4 ± 14.4	0.954^b^
Onset age, mean ± SD		25.8 ± 7.89	
Duration (years), mean ± SD		26.2 ± 13.2	

Abbreviation: SD, standard deviation.

^a^ *p*‐value was calculated using Fisher's exact test.

^b^ *p*‐value was calculated using Welch's *t*‐test.

**TABLE 2:** 5‐HTTLPR genotype and allele frequency of patients with schizophrenia and controls in this study.

			Genotype				Allele					
	*n*	HWE^a^	S/S	S/L	L/L	*p* ^b^	S	L	MAF	*p* ^c^	OR (95% CI)^d^	Power^e^
Overall										4,10		
SCZ	467	0.65	308	145	14	**0.007**	761	173	0.185	**0.007**	1.39 (1.08–1.77)	0.77
CTL	361	0.77	210	129	22		549	173	0.240			
Male												
SCZ	223	0.08	141	78	4	0.380	360	86	0.193	0.410	1.16 (0.80–1.68)	0.13
CTL	161	1.00	98	56	7		252	70	0.217			
Female												
SCZ	244	0.38	167	67	10	**0.006**	401	87	0.178	**0.005**	1.60 (1.14–2.24)	0.81
CTL	200	0.58	112	73	15		297	103	0.258			

*Note:* *p* < 0.05 is shown in bold.

Abbreviations: 5‐HTTLPR, serotonin‐transporter‐linked promoter region; CI, confidence interval; CTL, control; HWE, Hardy–Weinberg equilibrium; L, long allele; MAF, minor allele frequency; OR, odds ratio; S, short allele; SCZ, patients with schizophrenia.

^a^ *p*‐values for Hardy–Weinberg equilibrium calculated with Haldane's exact test.

^b^ Genotypic *p*‐values were calculated with the Cochran‐Armitage test.

^c^ Allelic *p*‐values were calculated with Fisher's exact test.

^d^ Odds ratio for schizophrenia on S allele.

^e^ Statistical power was estimated based on Cohen's h.

### 3.2 Meta‐Analysis

**FIGURE 1:**
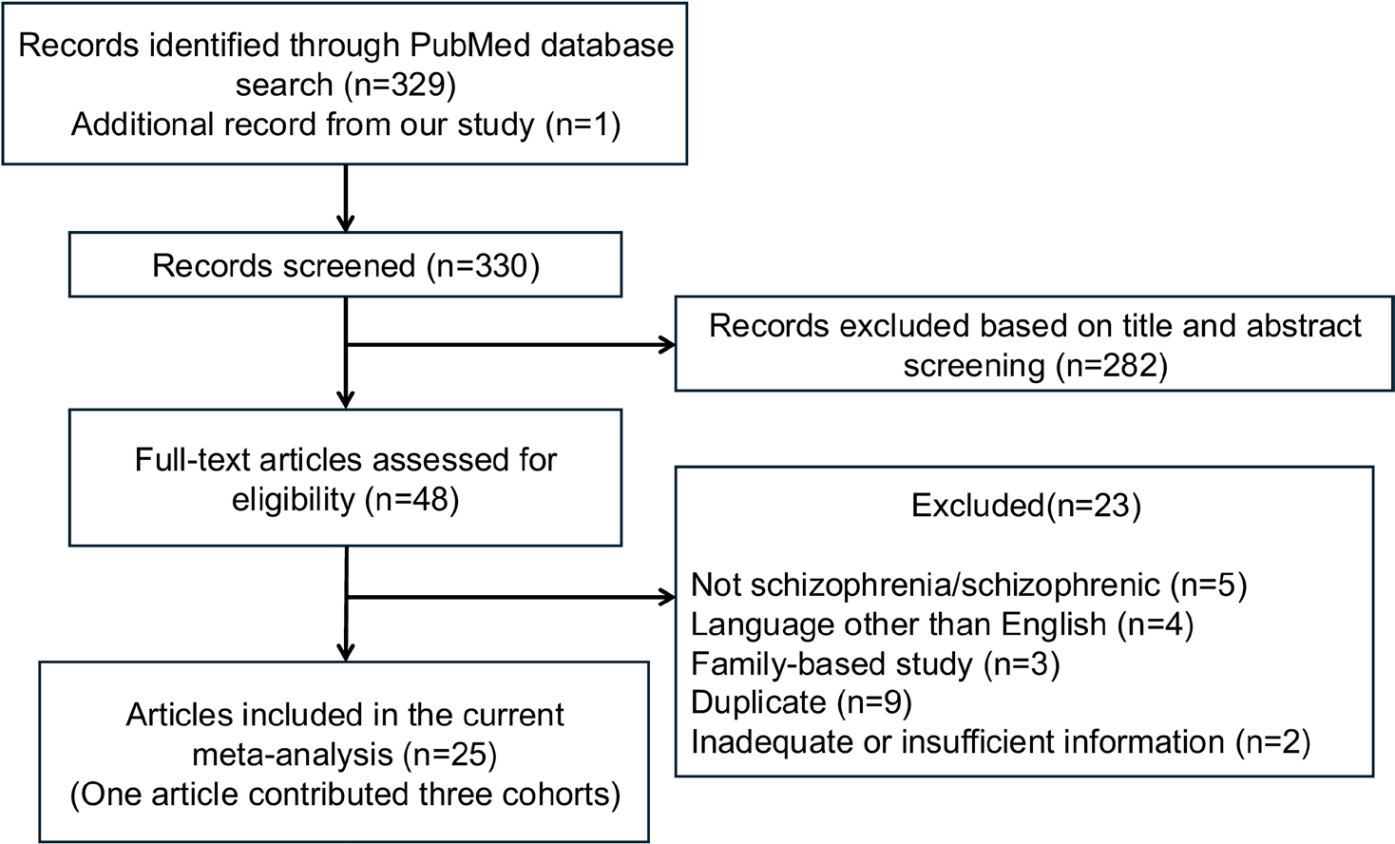
Flowchart of article selection for the meta‐analysis. Candidate articles were identified through PubMed using the search terms: (“serotonin transporter” OR “SERTPR” OR “SERT‐in2” OR “5‐HTTLPR” OR “SLC6A4”) AND (“schizophrenia”). A total of 25 studies (27 cohorts), including the present study, were included in the analysis.

**FIGURE 2:**
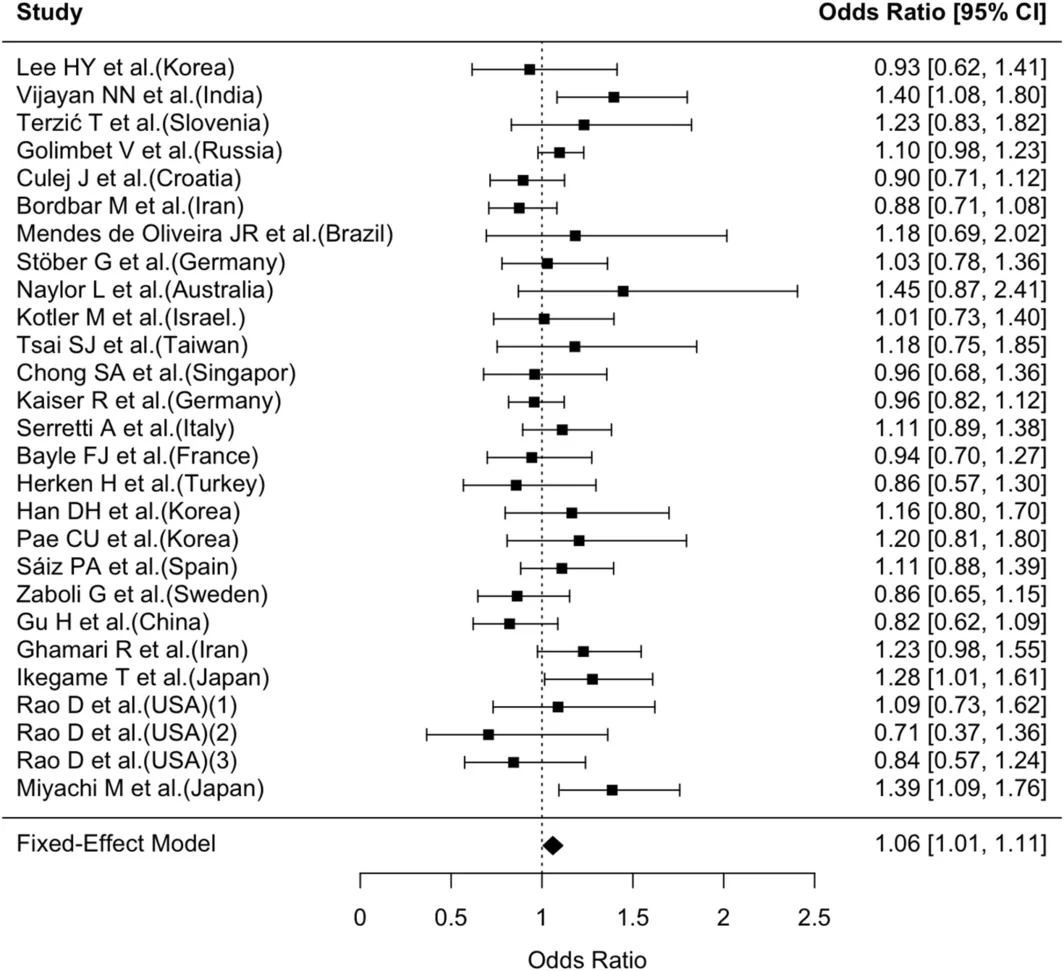
Forest plot of the meta‐analysis of the 5‐HTTLPR polymorphism and schizophrenia. The horizontal axis represents the odds ratios and 95% confidence intervals of short allele on schizophrenia. The vertical axis represents the first author of each study and the country where the cohort was recruited. We conducted this meta‐analysis with the R package metafor using a fixed‐effect model. (USA) (1) represents the cohort of US Caucasians, (2) represents US African Americans, and (3) represents Swedish Caucasians, respectively.

The literature search identified 25 eligible studies (27 cohorts), including the present study, for inclusion in this meta‐analysis (Figure 1). The results of the meta‐analysis are summarized as a forest plot in Figure 2. There is a significant association between the S allele frequency of 5‐HTTLPR and schizophrenia (OR = 1.06 [95% Cl = 1.01–1.11], *p* = 0.024). Between‐study heterogeneity was low (*I* ^2^ = 24.3%).

## 4 Discussion

This study demonstrated a significant association between the 5‐HTTLPR polymorphism and schizophrenia. In addition, the meta‐analysis also showed a significant association, but the effect size was smaller than that of our own cohort study. Prior research highlighted differences in genetic architecture across ethnic groups, including patients with schizophrenia [13, 14]. Especially, Japan is an island nation and may possess uniquely comprehensive genetic structures even among Asian populations [15]. The strong association observed in our study may be partly attributed to this unique genetic background of the cohort we examined.

Accumulating evidence supports the role of serotonin dysfunction in the pathophysiology of schizophrenia. Although the classical dopamine hypothesis has been central to understanding schizophrenia, the serotonin system, particularly the 5‐HT_2A_ receptor, has become increasingly recognized due to its contribution to hallucinations and delusions, as well as modulation of dopamine and glutamate systems [16, 17, 18]. Furthermore, psychiatric diseases closely related to schizophrenia, such as bipolar disorder and obsessive compulsive disorder, share overlapping symptoms and have also been reported to be associated with 5‐HTTLPR [9, 19, 20].

Study using structural magnetic resonance imaging has reported that the S allele is associated with reduced amygdala volume and increased amygdala activation [21]. Other suggested associations between *SLC6A4* variants and structural brain abnormalities among patients with schizophrenia [22] Dysregulation of the serotonin system and its complex interactions with dopamine and glutamate signaling are increasingly considered pivotal to schizophrenia pathogenesis [23]. This is consistent with the pharmacological development of atypical antipsychotics, which target serotonin receptors, including 5‐HT_2A_, in addition to dopamine receptors [24, 25, 26].

Genome‐wide association studies (GWAS) have identified numerous risk loci for schizophrenia; many of these loci require further investigation to elucidate their biological significance [27]. In this context, candidate gene association studies focusing on functionally well‐characterized variants remain valuable.

In the subgroup analyses of this study, results appeared to differ between males and females. Although both males and females showed a positive association between the S allele of 5‐HTTLPR and schizophrenia risk in this subgroup analysis, statistical significance was reached only in females, and the effect size was larger than in males. Previous studies showed that the concentration of serotonin in whole blood is higher in females than in males [28], and that the baseline serotonin function in females may also be elevated [29]. These biological differences could contribute to sex‐specific pathophysiological mechanisms and may help explain the sex‐related variability in schizophrenia observed in our study [30]. Very few prior studies have performed subgroup analyses stratified by sex. Accordingly, our observations regarding sex differences should be evaluated and validated together with future studies.

This study has several limitations. First, although all patients were diagnosed with schizophrenia using standard diagnostic criteria, detailed information on illness severity, treatment response, and mood or obsessive‐compulsive symptoms assessed by standardized instruments (e.g., HAM‐D or Y‐BOCS) was not available in this study. Second, we examined only the 5‐HTTLPR polymorphism, without addressing other variants that may influence *SLC6A4* expression. Third, the 5 HTTLPR polymorphism was analyzed using a biallelic S and L classification. Although a higher resolution classification incorporating rs25531 provides a more detailed functional interpretation of serotonin transporter activity, such information was not available in the present study [31]. As a result, our findings should be interpreted primarily at the level of genetic association rather than detailed functional stratification. Future studies incorporating rs25531 and other functional variants will be necessary to further refine the biological interpretation of the involvement of SLC6A4 in schizophrenia.

Despite these limitations, this study represents, to our knowledge, the cohort analysis including the largest sample size of Asian patients with schizophrenia to investigate the 5‐HTTLPR polymorphism. Our findings support a significant association between the S allele and schizophrenia risk. Further research is necessary to explore various genetic polymorphisms and molecular mechanisms in the serotonin system for the development of new treatments for schizophrenia.

## Author Contributions

**Masao Miyachi:** conceptualization, methodology, formal analysis, investigation, data curation, visualization, writing – original draft preparation. **Toshiyuki Shirai:** formal analysis, data curation, visualization, funding acquisition, writing – original draft preparation. **Shohei Okada:** methodology, formal analysis, investigation, data curation. **Kiriko Minami:** data curation. **Ryo Tsukamoto:** data curation. **Kentaro Mouri:** data curation. **Satoshi Okazaki:** conceptualization, methodology, funding acquisition, data curation. **Ikuo Otsuka:** conceptualization, methodology, funding acquisition, data curation. **Akitoyo Hishimoto:** conceptualization, funding acquisition, writing – review and editing, supervision, project administration.

## Funding

This work was partially supported by JST Moonshot R&D Program (Grant Number JPMJMS239F to Akitoyo Hishimoto and Ikuo Otsuka), JSPS KAKENHI (Grant Numbers JP25K19058 to Toshiyuki Shirai; JP21K07520, JP24K10710 to Satoshi Okazaki; JP24K10710, JP24K10732 to Ikuo Otsuka; JP21H02852, JP24K02383 to Akitoyo Hishimoto), Japan Agency for Medical Research and Development grants (Grant Number 22dk0307111 to Ikuo Otsuka), SENSHIN Medical Research Foundation (to Ikuo Otsuka), and the Smoking Research Foundation (to Akitoyo Hishimoto and Ikuo Otsuka).

## Ethics Statement

This study was approved by the Ethics Committee of the Graduate School of Medicine, Kobe University. In accordance with the Declaration of Helsinki, all participants were given sufficient written and verbal explanations about the study and provided their informed consent.

## Consent

All participants gave their consent to this study both verbally and in writing.

## Conflicts of Interest

The authors declare no conflicts of interest.

## Supporting information


**Table S1:** Detailed data of the participants.

## Data Availability

The data that supports the findings of this study are available in the Supporting Information of this article.
